# High Genetic Diversity With Weak Phylogeographic Structure of the Invasive *Spartina alterniflora* (Poaceae) in China

**DOI:** 10.3389/fpls.2019.01467

**Published:** 2019-11-20

**Authors:** Lei Shang, Lin-Feng Li, Zhi-Ping Song, Yi Wang, Ji Yang, Chuan-Chao Wang, Shi-Yun Qiu, Jing-Xin Huang, Ming Nie, Lorne M. Wolfe, Bo Li

**Affiliations:** ^1^Coastal Ecosystems Research Station of Yangtze River Estuary, Ministry of Education Key Laboratory for Biodiversity Science and Ecological Engineering, Institute of Biodiversity Science, Institute of Eco-Chongming (IEC), Fudan University, Shanghai, China; ^2^Institute of Forensic Science, Ministry of Public Security, Beijing, China; ^3^Ministry of Education Key Laboratory of Contemporary Anthropology, School of Life Sciences and Institutes of Biomedical Sciences, Fudan University, Shanghai, China; ^4^Key Laboratory of Biodiversity and Evolution, Fudan University, Shanghai, China; ^5^Department of Anthropology and Ethnology, Xiamen University, Xiamen, China; ^6^School of Energy and Environment Science, Yunan Normal University, Kunming, China; ^7^School of Life Sciences, University of KwaZulu-Natal, Scottsville, South Africa

**Keywords:** genetic admixture, mathematical simulation, population variation, plant invasion, rapid evolution, *Spartina alterniflora*

## Abstract

Biological invasion represents a global issue of concern due to its large negative impacts on native ecosystems and society. Elucidating the evolutionary history and genetic basis underpinning invasiveness is critical to understanding how alien species invade and adapt to novel environments. Smooth cordgrass (*Spartina alterniflora*, 2n = 6*x* = 62) is a notorious invasive species that causes heavily negative effects on native ecosystems worldwide. Here we addressed the evolutionary mechanisms underlying the invasion and dispersal history of this species along the China coast in the past decades. We employed nine microsatellites and three chloroplast fragments to investigate phylogeographic structure and genetic diversity of 11 native US and 11 invasive Chinese *S. alterniflora* populations. Demographic history simulation was also performed for both the native and invasive populations, respectively. Comparative genetic analyses of these natural populations revealed that although all the Chinese populations were introduced only once, high level of genetic diversity with weak geographic structure was observed. In particular, both the genetic features and mathematical simulation illustrated very recent population expansion in the Chinese populations. We found that genetic variants identified in native US populations were mixed in the Chinese populations, suggesting the recombination of these original variants during the invasion process. These genetic attributes indicate that Chinese populations might not have experienced a genetic bottleneck during the invasion process. High genetic diversity and genetic admixture might have contributed to the success of invasion of *S. alterniflora* in China. Our study provides a framework of how the smooth cordgrass spreads along the China coast as well as its potential genetic mechanisms underlying the invasion.

## Introduction

Invasive alien species (IAS) represent one of the greatest threats to global biodiversity due to their negative impacts on the functioning of native ecosystems (IUCN, 1999; Mack et al., 2000; Lee, 2002; Prentis et al., 2008; Vilà et al., 2011). Current strategies for IAS control and management include the use of chemical toxins and pesticides, classical biological control, and habitat removal (Myers et al., 2000; Courchamp et al., 2003; Harvey-Samuel et al., 2017). However, these deployments often cause significant off-target effects so that the IAS are only temporally mitigated rather than being eradicated from the local ecosystems (Simberloff, 2009; Zarnetske et al., 2010). To this end, further investigations focusing on the genetic basis of invasiveness can not only offer vital information for elucidating evolutionary mechanisms underlying the successful establishment of IAS, but also provide an efficient guideline for controlling and management of the IAS in the native ecosystems. Indeed, many attempts have been made to explain how the genetic variability contributes to the colonization of IAS to novel environments (Tsutsui et al., 2000; Kolbe et al., 2004; Hodgins et al., 2013; Ueno et al., 2015). For example, high level of genetic diversity is supposed to contribute high adaptability to IAS to cope with diverse stresses caused by natural selection (Ahlroth et al., 2003; Prentis et al., 2008; Wang et al., 2012; Hufbauer et al., 2013). Likewise, additional work by comparing the genetic differences between the native and invasive populations has also revealed the crucial roles of genetic variability in shaping life history and reproductive and defensive characters (Blair and Wolfe, 2004). These empirical studies support the notion that genetic variability is a critical determinant in the invasion process of IAS.

The genus *Spartina* (Poaceae) includes 13–15 species that all are perennial herbs and native to the Atlantic and northeastern Pacific coasts (Ainouche et al., 2009). Cytological studies indicate that all extant *Spartina* species are either tetraploid, hexaploid, or dodecaploid, most of which exhibit highly invading ability (Marchant, 1968; Ayres et al., 2008a; Ainouche et al., 2012). Among these polyploid species, *Spartina alterniflora* (2n = 6*x* = 62) is originally distributed in the Atlantic and Gulf coasts of North America but have become one of the most aggressive invaders in global coastal ecosystems (Wang et al., 2006; Strong and Ayres, 2013). In the United Kingdom (UK), for example, the invasive species *S. alterniflora* was accidentally introduced during the 19^th^ century (Ainouche et al., 2003). Homoploid hybridization between *S. alterniflora* and native *Spartina maritima* (2n = 6*x* = 60) gave rise to a sterile hexaploid hybrid *S. x townsendii*. Thereafter, chromosome doubling of the F1 hybrid of the two hexaploid species resulted in the formation of a fertile and high aggressive dodecaploid species *Spartina anglica* (2n = 12*x* = 100, 122, or 124) (Marchant, 1968). In the western coast of France, reciprocal crosses between the same two parental species had also led to the establishment of a morphologically distinct hexaploid species *Spartina x neyrautii* (Marchant, 1977). These recently formed polyploid species show relatively high tolerance to biotic and abiotic stresses so that they can occupy a wide range of habitats (An et al., 2007; Ainouche et al., 2009). Similar phenomenon has also been observed in the western coast of United States (US) where interspecific hybridization occurs frequently between the invasive *S. alterniflora* and native hexaploid hybrid *Spartina foliosa* (2n = 6*x* = 62) (Daehler and Strong, 1997). The resulting hybrids are genetically diverse and usually possess highly aggressive traits compared to their parental species in the salt marsh systems (Ayres et al., 1999; Ayres et al., 2003; Sloop et al., 2005; Ayres et al., 2008b).

In China, four *Spartina* species were introduced for the purpose of wetland restoration, tidal land reclamation, and saline soil mitigation (Zhang et al., 2004; Zuo et al., 2012). Of the four *Spartina* species, the dodecaploid *S. anglica* was introduced from England and Denmark in 1963 (Chung, 2006). Thereafter, the other three species, *S. alterniflora*, *Spartina patens* (2n = 4*x* = 40), and *Spartina cynosuroides* (2n = 4*x* = 40), were introduced from North American Atlantic coast in 1979 (Zuo et al., 2009; Zuo et al., 2012). To date, the three species, *S. anglica*, *S. patens*, and *S. cynosuroides*, are restricted to narrow ranges or almost disappeared from natural environments (An et al., 2007; Zuo et al., 2012). In contrast, the other species *S. alterniflora* has rapidly spread along the Chinese coast over the past decades and was officially listed as one of the top 16 invasive species in China (Wang et al., 2006; Zuo et al., 2012). These attributes offer the *S. alterniflora* an ideal system to address evolutionary mechanisms underlying the successful invasion of alien species. To this end, several previous studies attempted to elucidate how the genetic, morphological, and physiological factors contributed to the invasion of *S. alterniflora*. For example, historical investigation of the original sample collection records (both seeds and alive plants) revealed that all Chinese *S. alterniflora* populations were primarily originated from North Carolina, Georgia, and Florida of the US (Xu et al., 1989; Chen and Zhong, 1990; Bernik et al., 2016). While these original seeds of *S. alterniflora* were primarily transplanted in southeastern China, long-distance colonizing ability with fast growth rate has eventually resulted in a continuous distribution pattern across the coastal regions (Wang et al., 2006). In the US, high genetic diversity is observed in the native populations of *S. alterniflora*, especially most of the variants are explained by the differences among individuals within populations (Blum et al., 2007). As an invasive species originated through a single introduction, it is surprising that high genetic variability is observed in the Chinese populations (Wang et al., 2012; Bernik et al., 2016). However, these observations relied mainly on few samples and limited molecular markers.

The overarching goal of this study was to explore the evolutionary mechanisms underlying the introduction and subsequent invasion history of *S. alterniflora* in China. In this study, we collected a total of 1,227 accessions from 11 US native and 11 Chinese invasive populations. Based on this large sample size, we employed nine microsatellites and three chloroplast fragments to investigate the population structure and genetic variability between the US native and Chinese invasive populations. These genomic data allow us to evaluate if both the native and invasive populations show geographic structure and how these *S. alterniflora* populations spread along the China coast. In addition, we also performed a mathematical simulation to test whether or not the inter-population mixing among the three original populations contributed to the invasion success of *S. alterniflora* in China. Our study therefore provides an evolutionary perspective on how the invasive species *S. alterniflora* evolves in China.

## Materials And Methods

### Plant Materials and DNA Extraction

The species *S. alterniflora* possesses both sexual (self-compatible) and asexual reproductive strategies (Wang et al., 2006). Its seeds facilitate long-distance colonization through tidal water (Elsey-Quirk et al., 2009), and its rhizomes can promote strong clonal growth (Zhang et al., 2006). To obtain representative accessions from each wild population, two to five young leaves were sampled from each individual that spaced to each other at least 40 m apart. With this sampling strategy, we collected a total of 1,227 accessions from 11 US native and 11 Chinese invasive populations (**Supplementary Table 1**). In brief, the 11 invasive Chinese populations were collected along the coastline to represent its current distribution range in China. In addition, we also collected samples from 11 naturally distributed populations along the US eastern and southern coastline. Among the 11 native US populations, the 3 populations localized in North Carolina (U-MC), Georgia (U-SI), and Florida (U-TB) are believed to be the source of all Chinese *S. alterniflora* accessions (Chen and Zhong, 1990). Leaf samples were dried with silica gel and stored at −20°C. Genomic DNA was extracted from each accession using a Plant Genomic DNA Kit (Tiangen, Beijing, China).

### Microsatellite Genotyping and Chloroplast Fragment Sequencing

Previous studies have characterized a series of polymorphic microsatellites from *S. alterniflora* (Blum et al., 2004; Sloop et al., 2005). We tested the transferability of 35 microsatellites using 24 accessions collected from different populations. Nine of these microsatellite loci (SPAR.02, SPAR.05, SPAR.06, SPAR.07, SPAR.09, SPAR.10, SPAR.11, SPAR.20, SPAR.27) that generated clear bands were selected for genotyping in this study (**Supplementary Table 2**). PCR amplifications were performed in a volume of 10 µl including 0.2 µl of DNA (20–50 ng), 1 unit *Taq* DNA polymerase (Takara, Dalian, China), 0.8 µl of dNTPs, 0.3 µl of each primer (10 µM), 1 µl of 10× PCR buffer (Mg^2+^ included), and 7.3 µl ddH_2_O. The forward primer for each primer pair was fluorescently labeled with FAM, HEX, or ROX (**Supplementary Table 2**). The PCR reactions were conducted on an ABI 9700 thermocycler: an initial denaturation of 5 min at 94°C, 30 cycles of 30 s at 94°C, annealing temperature (Ta) for 30 s (Ta for each primer pair is listed in **Supplementary Table 2**), 30 s at 72°C, and a final extension of 72°C for 5 min. The labeled PCR amplicons were then sized against ABI GS500LIZ standard on an ABI 3730 automated sequencer and scored with GeneMapper v.4.0 (Applied Biosystems, California, USA). As a complementary step, we also sequenced three chloroplast fragments (*trnT2-rps4*, *trnT-L*, and *rbcL-psaI*) of 207 representative samples, of which 92 and 115 accessions were selected from the USA and Chinese populations, respectively (**Supplementary Table 1**). All PCRs were carried out in a thermal volume of 50 µl: 2 µl of genomic DNA, 0.25 µl of Taq polymerase (Takara, Dalian, China), 4 µl of dNTP, 2 µl of each primer, and 5 µl of 10× buffer. PCR amplifications were generated by an ABI thermal cycler following a regime of one cycle at 94°C for 2 min, followed by 35 cycles of 94°C for 1 min, Ta (55°C for the *trnT-trnL* and 56°C for the *trnT2-rps4* and *rbcL-psaI*) for 1 min, 72°C for 2 min, and a final extension at 72°C for 5 min. All PCR amplicons were then purified and sequenced using an ABI 3730 automated sequencer (Applied Biosystems, USA).

### Data Correction, Population Genetic, and Phylogeographic Analyses

The program Microchecker v.2.2.3 (van Oosterhout et al., 2004) was used to correct the null allele in the microsatellite matrix. In addition, the species *S. alterniflora* can spread rapidly through its rhizomes. We therefore eliminated the data bias caused by the asexual reproduction based on the clonal diversity (the number of genotypes/sample size). A total of 173 accessions with genotype occurring more than once were excluded from the data matrix. To further minimize the effects of sample size on genetic indices, these populations with relatively large sample size (>43 samples) were recollected. To further explore the tendency that genetic diversity of native populations might vary with sample size, we performed a simulation of sampling, of which individuals from US populations were randomly sampled along a gradient of sample sizes: 2, 5, 25, 50, 100, 200, and 400. Ten replicates were conducted for each simulation for a given sample size. We also calculated Shannon’s information index (*I*) for each sampling and determined the proportion of *I* (*I* for the sampled population/*I* for US populations as a whole). The fitted curve was then added after a simulation of nonlinear model in the software R v.3.0.2 (R Core Team, 2013). Related computer source codes are given in **Supplementary Text 1**.

The corrected microsatellite data set was subjected to subsequent analyses of genetic diversity and population structure. Hardy–Weinberg equilibrium was tested for each microsatellite using Genepop v.4 (Rousset, 2008). It has been suggested that all Chinese *S. alterniflora* populations were introduced from the three US populations (U-MC, U-SI, and U-TB). To this end, we defined the 22 Chinese and US populations as three groups: (1) the source group containing the 3 USA populations (U-MC, U-SI, and U-TB) that were supposed to be the ancestors of all Chinese populations; (2) the non-source group including all the other 8 native USA populations; and (3) the introduced group that consisted of all the 11 Chinese populations (**Supplementary Table 1**). The software GenAlex v.6.5b3 (Peakall and Smouse, 2006; Peakall and Smouse, 2012) was employed to calculate the genetic diversity parameters at both the population and group levels, including the number of effective alleles (*Ae*), Shannon’s information index (*I*), individual inbreeding coefficient (*Fi*), and private allele (*Pa*). To further assess the genetic variation pattern within and between populations, analysis of molecular variation (AMOVA) and principal coordinate analysis (PCoA) were performed for all 22 populations using Arlequin v.3.5.1.2 (Excoffier et al., 2005). We also estimated genetic assignments of the 22 Chinese and US populations using STRUCTURE v.2.3.4 (Pritchard et al., 2000). The *K* values were calculated from 1 to 23 with 20 independent iterations. Each run was executed using the admixture model with an initial burn-in period of 10^4^ and followed by 10^5^ Markov Chain Monte Carlo (MCMC) iterations, and the delta *K* was used to determine the best genetic assignment.

Chloroplast sequences were checked against the original graphic files and aligned using MEGA v.5 (Tamura et al., 2011) with correction if necessary. Sequences of the three chloroplast fragments were combined as a single matrix for subsequent data analyses. In detail, haplotypes were identified on the basis of variable sites (including both of the single nucleotide polymorphism and insertion/deletion). Haplotype network was constructed using the program NETWORK v.4.6 (http://www.fluxus-engineering.com;Bandelt et al. 1999). Phylogeographic structure was tested for both native and introduced groups by comparing the values of Gst and Nst using Haplonst (Raymond and Rousset, 1995) and U-statistics (Pons and Petit, 1996). To further reveal the geographic distribution of these chloroplast haplotypes, we calculated the number of haplotypes within each of the 22 populations. As a complementary of the above microsatellite data analyses, we also estimated the average gene diversity at both the population (HS) and group (HT) levels using Haplonst (Raymond and Rousset, 1995). In addition, haplotype diversity (Hd), nucleotide diversity (π), and AMOVA were also calculated using Arlequin v.3.5.1.2 (Excoffier et al., 2005).

### Demographic History Simulation

Theoretically, invasive species are expected to show relatively low genetic diversity due to the bottleneck effect during the invasion process. To experimentally test whether the invasive populations found in China have undergone a genetic bottleneck, we used the microsatellite data set to simulate the effective population size for both the native and introduced populations using Bottleneck v.1.2.02 (Cornuet and Luikart, 1996), with the infinite alleles model (IAM), stepwise mutation model (SMM), and two-phased model (TPM). Both the sign and Wilcoxon sign rank tests were applied to assess the deviation from mutation-drift equilibrium. In addition, we also applied the Bayesian evolutionary analysis by sampling trees (BEAST) to estimate the effective population size changes based on the chloroplast data set (Drummond et al., 2002; Drummond and Rambaut, 2007), with the HKY substitution model without I+G and strict clock (Richardson et al., 2001). The piecewise-linear skyline coalescence model was chosen with the number of groups (bins) setting as 10. Single MCMC chain was run for 20 million steps and sampled for every 1,000 steps. The log file was inspected in tracer for convergence of the chain and the effective sample size (ESS) values, and the Bayesian skyline reconstruction was run. Using the Bayesian skyline plots generated from BEAST, we calculated the rate of population growth per year. Each skyline plot consisted of 100 smoothed data points. The initial population size was set as the minimum population size during the period immediately preceding population growth. We also estimated the population growth rate for the interval in our data where the growth was the fastest. We chose the exponential growth equation for this analysis as suggested in Gignoux et al. (2011): *r*=ln(*Nt*/*N* *_0_*)/*t*, where *r* represents the population growth rate per year, *N* *_0_* is the initial population size, and *t* is the time since growth began. To further estimate the genetic contribution rate from the native populations to the introduced populations, we used *a*, *b*, *(1* – *a* − *b)* to respectively represent the relative genetic contributions of the 3 source populations (U-MC, U-SI, and U-TB) to all the 11 Chinese populations. The probability obtained from observed alleles and their frequencies in Chinese populations was calculated as follows:

(1) $$P=\prod [a\times f_{ij,MC}+b\times f_{ij,SI}+(1-a-b)\times f_{ij,TB}{]}^{n_{ij,China}}$$

where *i* ranges from the first microsatellite locus SP02 to the ninth locus SP27; *j* ranges from the first allele to the last allele for each locus; *f* *_ij,MC_*, *f* *_ij,SI_*, and *f* *_ij,TB_* represent the frequencies of allele *j* at locus *i* in populations U-MC, U-SI, and U-TB, respectively; and *n* *_ij,China_* represents the number of allele *j* at locus *i* in Chinese populations. With the feasibility of calculation considered, we applied the logarithmic transformation to equation (1). We then obtained:

(2) $$\text{ln}(P)=\sum n_{ij,China}\times \ln[a\times f_{ij,MC}+b\times f_{ij,SI}+(1-a-b)\times f_{ij,TB}]$$

A grid search was then conducted to find the optimal solution to equation (2), with *a*, *b*, and *(1* – *a* − *b)* ranging from 0 to 1. To further simulate population genetic mixing, we explored the effect of admixture on amount of genetic variation with the assumption that the three native source populations were mixed evenly. Thus, *N* was taken as the original sample size for each native source population. We then conducted a simulation of genetic mixing to produce observed alleles frequencies (where expected = admixture from three source populations and observed = Chinese) and then generated the optimal value of *N*. Based on allele frequency data of native source populations estimated above, we next calculated the expected distribution of alleles at the nine microsatellite loci for Chinese populations based on the formulas *a*×*2N* for U-MC, *b*×*2N* for U-SI, and (*1* – *a* − *b*)×*2N* for U-TB, respectively. All these simulations were performed using the approximate Bayesian computation (ABC), with the times of repetition for each *N* being set to 1 billion. The computer codes for simulation are given in the **Supplementary Text 2**, which were run directly on LINUX operating system.

## Results

### Genetic Diversity and Variation Pattern

All nine microsatellite markers used in this study were successfully amplified across all the 1,227 samples. As the *S. alterniflora* is an allohexaploid species, we examined whether or not the null alleles existed in the microsatellite matrix. At the population level, all the 22 populations showed different degrees of null alleles in the nine microsatellites, with the Chinese population C-NF only possessing null alleles in the locus SP27 but the source US population U-SI having null alleles across all microsatellite markers (**Supplementary Table 3**). At the locus level, the nine microsatellites harbored null alleles, ranging from 5 populations at the locus SP20 to 20 populations at the locus SP27 (**Supplementary Table 3**). While the above statistics identified null alleles in the microsatellite matrix, most of which exhibited low null allele frequency (<0.2) across the data matrix. To eliminate data bias in subsequent data analyses, all these detected null alleles were corrected at each locus. In addition, we also assessed clonal index to examine the effects of asexually reproductive strategy on the estimation of genetic diversity. Our results revealed that while the clonal diversity varied among the 22 populations, both US (average value = 0.87) and Chinese (average value = 0.85) groups showed similar level of clonal diversity (**Table 1**). To further test whether the sampling size affected the assessment of genetic diversity, we simulated the Shannon’s information index with different numbers of sampling size. The variation tendency of the proportion of the Shannon’s information index reached saturation curve very fast (sampling size < 25) (**Supplementary Figure 1**). These features suggest that the estimation of genetic diversity was not significantly affected by the difference in sampling size.

**Table 1:** Measures of clonal and genetic diversity of native and invasive *Spartina alterniflora*.

Region	Site code	*Ha*	*Hd*	*Pi* (×10^−3^)	Clonal diversity	*Ae*	*I*	*Ho*	*He*	*Fi*
**United States**	U-MC †	2	0.485	0.810	0.90	6.210	1.918	0.682	0.810	0.146**
	U-SI †	3	0.689	0.978	0.96	5.708	1.877	0.648	0.805	0.180**
	U-TB †	2	0.409	0.228	0.25	2.835	1.151	0.544	0.627	0.100**
	U-NE	2	0.530	0.886						
	U-SV	1	0.000	0.000						
	U-CC				0.92	2.492	0.982	0.428	0.520	0.151**
	U-RI				0.93	2.831	1.040	0.354	0.523	0.243**
	U-LS				0.86	3.067	1.204	0.496	0.607	0.151**
	U-DJ	2	0.429	0.716	0.98	4.886	1.657	0.564	0.778	0.250**
	U-TP	4	0.644	0.681	0.94	3.415	1.393	0.605	0.671	0.092**
	U-GV	4	0.867	0.669	1.00	3.311	1.270	0.483	0.679	0.275**
	U-BR	1	0.000	0.000	0.96	2.180	0.971	0.315	0.520	0.355**
	U-ML	2	0.533	0.594						
	U-FP	1	0.000	0.000	0.86	4.196	1.488	0.614	0.756	0.161**
Mean ± SD						3.739	1.359	0.521	0.663	0.191
						1.337	0.340	0.119	0.114	0.080
US Populations		10	0.794	1.250	0.87	6.614	2.020	0.565	0.810	0.300**
Mean ± SD						4.917	1.648	0.625	0.747	0.142
						1.821	0.431	0.072	0.104	0.041
US Source Populations		5	0.752	1.041	0.76	7.005	2.050	0.657	0.825	0.200**
**China**	C-TH	1	0.000	0.000	0.91	1.803	0.641	0.304	0.403	0.235**
	C-TJ	2	0.485	0.810	0.99	4.129	1.610	0.641	0.744	0.124**
	C-DY	1	0.000	0.000	0.96	3.295	1.309	0.534	0.691	0.215**
	C-LY	2	0.509	0.851	1.00	3.280	1.319	0.574	0.693	0.154**
	C-YC	2	0.533	0.891	0.96	3.888	1.566	0.630	0.731	0.126**
	C-CM	2	0.556	0.929	0.85	4.227	1.491	0.584	0.723	0.163**
	C-WL	2	0.467	0.780	0.93	2.729	0.980	0.462	0.544	0.137**
	C-NH	1	0.000	0.000	0.84	2.313	0.919	0.409	0.520	0.121**
	C-NF	3	0.644	0.829	0.91	3.599	1.383	0.556	0.683	0.124**
	C-ZH	2	0.556	0.929	0.60	2.486	1.070	0.583	0.563	−0.024**
	C-ZJ	2	0.356	0.396	0.28	2.516	0.909	0.426	0.510	0.049**
Mean ± SD						3.115	1.200	0.519	0.619	0.129
						0.801	0.314	0.105	0.114	0.071
Chinese Populations		3	0.591	0.932	0.85	4.685	1.728	0.532	0.745	0.282**

Ha, number of chloroplast haplotype; Hd, haplotype diversity; Pi, nucleotide diversity; Ae, number of effective alleles; I, Shannon’s information index; Ho, observed heterogeneity; He, unbiased expected heterogeneity; Fi, individual inbreeding coefficient; **, p < 0.01, for significant deviation from Hardy–Weinberg equilibrium. † Native source populations from which S. alterniflora was introduced to China in 1979.

Based on the corrected microsatellite data matrix, we calculated the genetic diversity parameters at both the population and group levels. At the population level, the number of mean *Ae* varied from 1.803 in the Chinese population C-TJ to 6.210 in the US population U-MC (**Table 1**). At the group level, compared to the 11 Chinese populations (*Ae* = 4.685), the 11 US populations (*Ae* = 6.614) harbored relatively higher genetic diversity, and this trend was more pronounced in the 3 US source populations (*Ae* = 7.005). Similar phenomena were also observed in the other three genetic diversity parameters (*I*, *Ho*, and *He*) where the population C-TJ and U-MC harbored the lowest and highest genetic diversity among the 22 populations, respectively; and the US group showed relatively high genetic diversity compared to the Chinese group (**Table 1**). Through analyzing the individual inbreeding coefficient value (*Fi*), all these samples were found to show significant deviation from Hardy–Weinberg equilibrium at both the population and group levels. For the three chloroplast fragments, a total of nine sequence polymorphisms corresponding to 10 haplotypes were identified across the 207 individuals (**Supplementary Table 4**). All the 10 haplotypes were identified in the US populations, but the Chinese population only possessed 3 haplotypes (**Table 1**). The level of genetic diversity (*Hd* and π) varied among the 22 populations, with 6 populations (U-SV, U-BR, U-FP, C-TH, C-DY, and C-NH) showing no sequence polymorphisms and the populations U-GV (Hd = 0.867) and U-SI (π = 0.978 × 10 ^−3^) harboring higher Hd and π values (**Table 1**). Being consistent with above microsatellite data set, the overall 11 US populations (Hd = 0.794, π = 1.250 × 10^−3^) exhibited relatively higher genetic diversity compared to all the 11 Chinese populations (Hd = 0.591, π = 0.932 × 10^−3^) (**Table 1**).

### Population Genetic and Phylogeographic Structure

**Figure 1:**
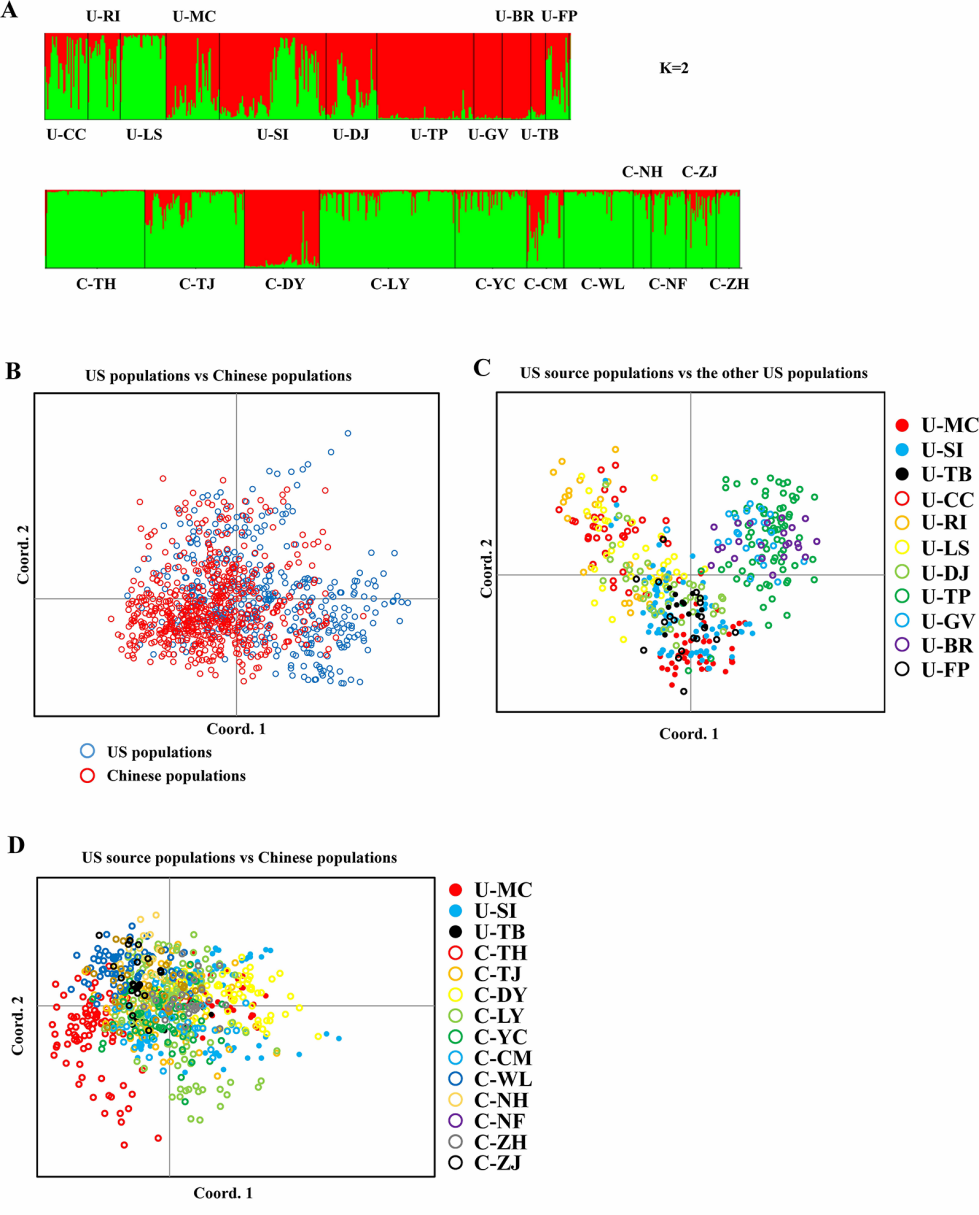
*Spartina alterniflora* populations. **(A)** Bar plot estimated by STRUCTURE representing assignments of genotypes of *S. alterniflora* from all populations to each cluster (K = 2). **(B)** PCoA of *S. alterniflora* between US and Chinese populations. **(C)** PCoA of *S. alterniflora* between US source populations and the other US populations. **(D)** PCoA of *S. alterniflora* between US source populations and Chinese populations.

Bayesian genetic assignments revealed that the three northeastern US populations possessed a genetic cluster (mostly in green color) that is different from the four Caribbean populations (mostly in red color), and the remaining four populations exhibited a mixed genetic structure (**Figure 1A**). In contrast, while both genetic clusters (red and green colors) were shared between the US and Chinese populations, 9 of the 10 Chinese populations showed basically identical genetic assignments (mostly in green color) (**Figure 1A**). To further examine this genetic structure, we performed PCA analysis at both the population and group levels. Being consistent with the results of Bayesian genetic assignments, no obvious genetic divergence was found between the US and Chinese groups (**Figure 1B**). At the population level, moderate genetic divergence was observed between the four northeastern and three Caribbean populations (**Figure 1C**). In contrast, no genetic divergence was found between the source US populations and Chinese populations (**Figure 1D**). We also estimated the distribution pattern of the 22 populations at both the population and group levels. As revealed by the AMOVA, 80.83–92.74% of the variances were explained by the within-population variation for the source US native, non-source US native, Chinese invasive, and overall populations (**Table 2**). In contrast, 7.26–17.44% of the total variance was accounted by the among-population variation. In addition, we found that only 2.37% of the variance was from the between US and Chinese groups, being consistent with above genetic assignment and PCA results that no clearly genetic divergence is found between the US and Chinese populations.

**Table 2:** Results of AMOVA showing distribution of genetic variation of *S. alterniflora* in US and China based on microsatellite dataset.

Source of variation	d.f.	Sum of squares	Percentage of variance explained
All populations combined (n=22)			
Between ranges	1	129.465	2.37%
Among population within ranges	20	949.136	16.80%
Within populations	1,020	4,690.337	80.83%
Total	1,041	5,768.938	
Native populations only (n=11)			
Among populations	10	383.196	16.78%
Within populations	412	1,905.709	83.22%
Total	422	2,288.905	
Native source populations only (n=3)			
Among populations	2	39.981	7.26%
Within populations	138	765.161	92.74%
Total	140	805.143	
Invasive populations only (n=11)			
Among populations	10	565.94	17.44%
Within populations	608	2,784.628	82.56%
Total	618	3,350.568	

**Figure 2:**
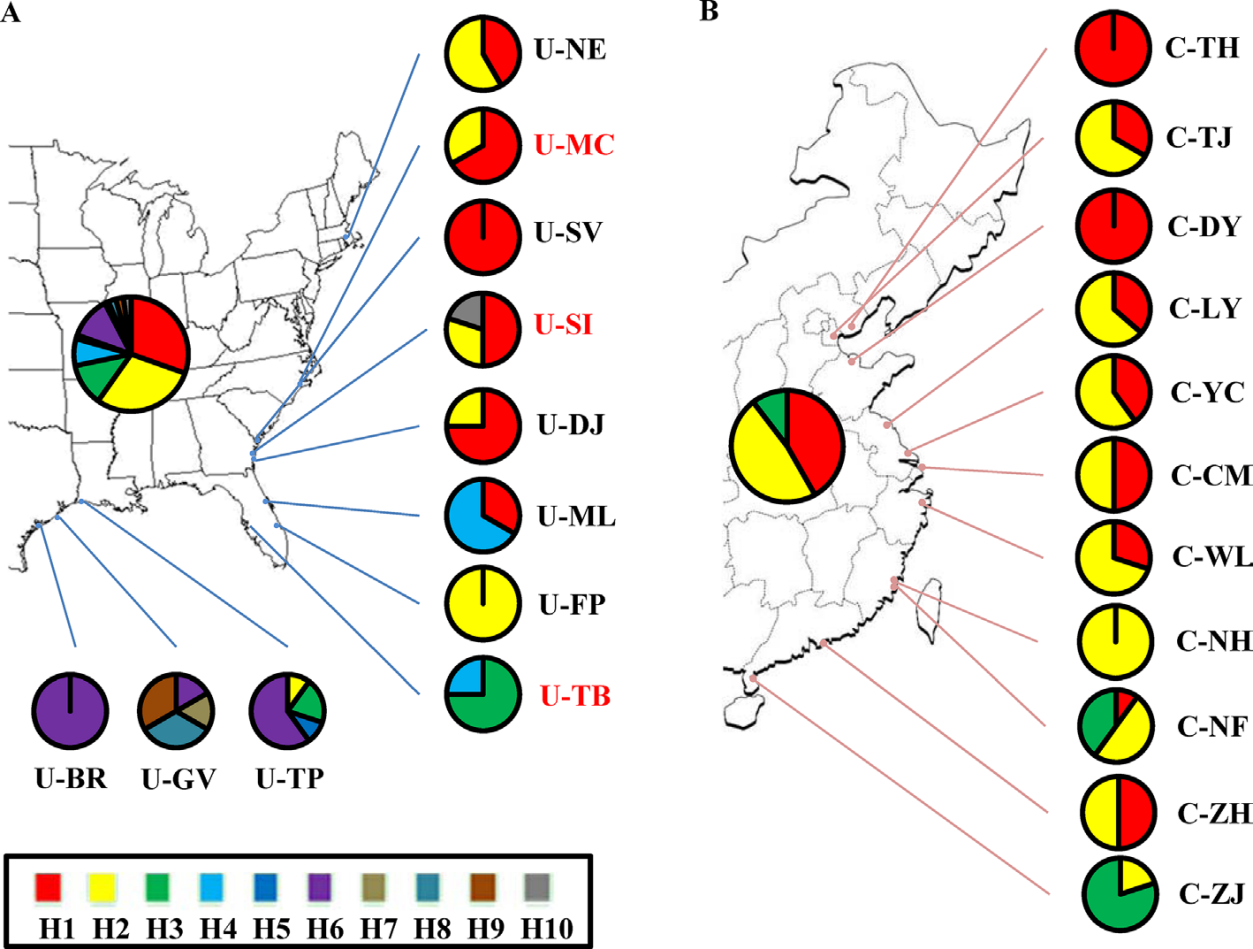
Geographic distribution of 10 chloroplast haplotypes detected in *S. alterniflora*. Pie charts represent the proportion and distribution of haplotypes across **(A)** native US and **(B)** invasive China ranges. Population and haplotype names in the map are the same as those in **Table 1** and **Supplementary Figure 2**.

Although the above-population genetic analyses revealed moderate genetic differentiation between northern US and Caribbean populations, no significant phylogeographic structures were found within either of the US and Chinese groups (U-statistics, *p* > 0.05). However, the haplotype network showed that seven minor haplotypes (H4-H10) were specific to the US populations, whereas the three major haplotypes were shared between the US and Chinese groups (**Supplementary Figure 2**). We also examined the correlations between the 10 chloroplast haplotypes and their geographic distributions. In the US group, the haplotypes found in the northeastern US populations were highly different from the Caribbean populations (**Figure 2A**). In the Chinese group, a weak correlation between the haplotypes and geographic distribution was also found, with the northern and southern populations mainly consisting of the H1 and H3 haplotypes respectively, and the H2 haplotype widely distributed across the China coast (**Figure 2B**). Comparing the 11 Chinese populations with the 3 source US populations revealed that the haplotype H1 was found to exhibit a relatively higher proportion in the northern populations and the haplotype H3 was specific to the southern populations (**Figures 2A, B**). Unlike the microsatellite data set, percentages of variances explained by the among-population variation (39.58–43.86%) were slightly lower than by within-population variation (56.14–60.42%) (**Table 3**). In addition, the genetic differentiation between populations was higher in the chloroplast fragments (F_ST_ = 0.400–0.439) compared to the microsatellite data set (F_ST_ = 0.070 – 0.170) (**Tables 2** and **3**).

**Table 3:** Analysis of molecular variance for populations of *S. alterniflora based on chloroplast dataset*.

Source of variation	d.f.	Sum of squares	Percentage of variance explained	F-statistics
All populations combined (n=22)				
Between ranges	1	1.761	19.00%	F_CT_=0.002
Among population within ranges	20	31.788	41.78%	F_ST_=0.420
Within populations	185	38.045	58.03%	F_SC_=0.419
Total	206	71.585		
Native populations only (n=11)				
Among populations	10	17.286	43.86%	F_ST_=0.439
Within populations	81	18.833	56.14%	
Total	91	36.120		
Invasive populations only (n=11)				
Among populations	10	14.492	39.58%	F_ST_=0.400
Within populations	104	19.212	60.42%	
Total	114	33.704		

### Demographic History and Colonization Route

We simulated how the *S. alterniflora* spreads in China and whether it experienced a genetic bottleneck during the invasion process. The simulation of the bottleneck effect based on microsatellite data set indicated that none of the 22 native and invasive populations has experienced significant bottleneck across three models (**Table 4**). We also estimated contributions of the 3 source US populations to the 11 Chinese populations. Results from the ABC simulation revealed a bell-shaped tendency for matching frequency at different sample sizes (**Supplementary Table 5**). The optimal sample size of 61 showed the highest matching frequency for each native source population, indicating that only a small sample size could result in the genetic diversity of current Chinese populations (**Supplementary Table 5**). Based on the simulation results, we found that the US source population U-SI contributed 76% of genetic constitution to the Chinese populations, which was obviously higher than those of U-MC (22%) and U-TB (2%). To further address the colonization history of these Chinese populations, we identified a total of 20 private alleles that were present in the three source US populations but absent in the other US populations (**Supplementary Table 6**). Analyzing the distribution pattern of these private alleles in the Chinese populations, we found that five accessions of the population C-YC have novel combinations (genotypes) of these private alleles (**Supplementary Table 6**), which suggested the possibility of admixture history in the Chinese populations. To assess whether or not the native US and invasive Chinese groups had undergone genetic bottleneck in the long-term evolutionary process, we estimated the effective population size for each of the two groups and at the whole species level. Our Bayesian simulations revealed that the native US group as well as the total species experienced a rapid increase in effective population size during the past 1,500 and 2,500 years (**Supplementary Figures 3A, B**). In contrast, the curve of the Chinese populations stretched very slowly, suggesting that *S. alterniflora* did not experience population expansion until 35 years ago (**Supplementary Figure 3C**). In addition, we also found that the growth rate of Chinese populations (growth rate = 0.02) was much higher than those of the native US group (growth rate = 0.0005) and the whole species (growth rate = 0.0016) (**Supplementary Figure 4**). It was broadly matched with the rapid invasion history of *S. alterniflora* in China.

**Table 4:** Sign test and Wilcoxon sign rank test of bottleneck estimates in *S. alterniflora* populations.

Region	Population	Sign test						Wilcoxon sign rank test		
		IAM		SMM		SMM		IAM	TPM	SMM
		*H* *_E_* */H* *_D_*	*P*	*H* *_E_* */H* *_D_*	*P*	*H* *_E_* */H* *_D_*	*P*	*P*	*P*	*P*
**United**	U-MC †	6/3	0.487	5/4	0.519	3/6	0.108	0.082	0.455	0.898
**States**	U-SI †	6/3	0.492	4/5	0.269	3/6	0.109	0.102	0.820	0.990
	U-TB †	2/7	0.036*	2/7	0.031*	2/7	0.018*	0.898	0.936	0.997
	U-CC	2/7	0.048*	1/8	0.011*	1/8	0.008**	0.986	0.993	0.993
	U-RI	1/8	0.006**	1/8	0.005**	1/8	0.004**	0.993	0.997	0.998
	U-LS	5/4	0.549	4/5	0.284	2/7	0.027*	0.545	0.850	0.990
	U-DJ	9/0	0.009**	8/1	0.071	5/4	0.552	0.001**	0.005**	0.715
	U-TP	4/5	0.288	4/5	0.287	3/6	0.102	0.455	0.875	0.986
	U-GV	8/1	0.050	7/2	0.216	6/3	0.453	0.064	0.082	0.285
	U-BR	2/7	0.032*	2/7	0.028*	1/8	0.003**	0.981	0.997	0.999
	U-FP	8/1	0.061	5/4	0.556	5/4	0.558	0.003**	0.082	0.455
**China**	C-TH	6/3	0.295	5/4	0.623	3/6	0.123	0.285	0.590	0.976
	C-TJ	7/2	0.237	3/6	0.110	0/9	0.000**	0.010*	0.898	1.000
	C-DY	8/1	0.056	6/3	0.452	3/6	0.101	0.002**	0.125	0.898
	C-LY	6/3	0.453	4/5	0.281	1/8	0.004**	0.024*	0.850	0.997
	C-YC	7/2	0.227	3/6	0.100	0/9	0.000**	0.082	0.918	1.000
	C-CM	8/1	0.051	8/1	0.061	5/4	0.569	0.007**	0.064	0.285
	C-WL	8/1	0.031*	7/2	0.159	6/3	0.416	0.003**	0.005**	0.082
	C-NH	3/6	0.142	3/6	0.123	3/6	0.135	0.820	0.918	0.981
	C-NF	6/3	0.474	2/7	0.027*	0/9	0.000**	0.367	0.990	1.000
	C-ZH	5/4	0.573	3/6	0.112	3/6	0.103	0.590	0.898	0.990
	C-ZJ	6/3	0.309	6/3	0.381	6/3	0.427	0.213	0.455	0.545
	China	9/0	0.010*	3/6	0.113	0/9	0.000**	0.001**	0.850	1.000

IAM, infinite alleles model; TPM, two-phased model; SMM, stepwise mutation model; H_E_/H_D_, ratio of number of locus with heterozygote excess against deficiency; *, P<0.05; **, P<0.01. † Native source populations from which S. alterniflora was introduced to China in 1979.

## Discussion

### Demographic and Invasion History of Chinese *S. alterniflora* Populations

*S. alterniflora* is a worldwide invasive species that possesses several mechanisms to enhance salt tolerance (e.g., ion exclusion at root level and secretion in leaves through salt glands), all of which confer it exceptionally high adaptability to salinity and tidal submersion (Bradley and Morris, 1991; Daehler and Strong, 1997; Bedre et al., 2016). Therefore, it is not only a dominant salt marsh grass in its native range of Atlantic and Gulf coasts of the North America but also a successful colonizer in the other coastal regions as an invasive species (Ainouche et al., 2012). In case of China, *S*. *alterniflora* has spread very fast along the coast over the past 40 years, whereas all current invasive Chinese populations are originally planted in the Luoyuan town of Fujian province (Southeastern China) (Xu et al., 1989; Wang et al., 2006). In this study, our results from both the microsatellite and chloroplast data sets revealed weak geographic structure between the northeastern Atlantic and Caribbean *S*. *alterniflora* populations, being broadly consistent with a previous study that found the two geographic groups to be genetically diverged from each other (Blum et al., 2007). This phylogeographical structure observed between the two native geographic groups can be explained by either distinct environmental conditions or demographic histories. As expected, our demographic simulations revealed very recent population expansion (∼2,500 years) of *S*. *alterniflora* in North America. Given that the southern Atlantic coast of US (e.g., Florida) is possibly an independent refugee during the last glacial maximum, we propose that distinct recolonization routes of the two geographic groups might be partially responsible for the observed genetic divergence. In addition, environmental differences between the local habitats as well as migration barriers are also potential factors that might have contributed to the observed geographic patterns (Blum et al., 2007).

The three source US populations (U-MC, U-SI, and U-TB) showed mixed genetic structure, and most of the genetic variability was identified in the Chinese populations. This observation is further confirmed by the demographic simulation that no significant genetic bottleneck was found in the Chinese populations. Instead, obvious recent population expansion with high growth rate was detected in these invasive populations, which is consistent with the rapid invasion history of *S*. *alterniflora* in China. This demographic attribute is different from that observed in *S*. *alterniflora* of the US west coast as well as some other invasive species, in which founder effect and genetic bottleneck are common features during the colonization and range expansion process (Meimberg et al., 2006; Blum et al., 2007; Zhang et al., 2010). High genetic diversity of invasive species is possibly due to the multiple introductions or admixture genetic stock of the source populations (Genton et al., 2005; Kelager et al., 2013). In our case, all the Chinese populations were introduced only once and primarily planted together in the Fujian province of southeastern China (Xu et al., 1989). To this end, while the three source US populations showed differentially genetic contributions, it is more likely that high genetic diversity observed in the Chinese invasive populations is possibly because of admixture of the independent genetic stocks of the three US source populations. With regard to the invasive routes of *S*. *alterniflora*, the chloroplast network analyses revealed weak geographic structure among these Chinese populations, with the northern Chinese populations possessing chloroplast haplotypes highly similar to the two northern source US populations (U-MC and U-SI) and the southern Chinese populations harboring the unique haplotype identified in the U-TB population. Of significance, the two southern invasive populations, C-NH and C-NF, are geographically very close to the primary transplanting location. These findings suggest independent dispersal routes of the northern and southern Chinese populations during the invasion process.

### High Genetic Diversity Followed By Admixture of Independent Genetic Stocks Enhances the Species Invasiveness

The importance of high genetic diversity to the successful establishment of alien species has been well recognized in many different invasive species (Prentis et al., 2008; Wang et al., 2012; Hufbauer et al., 2013). As reported in the European and western US coastal regions, hybridization between the invasive *S*. *alterniflora* and native congeneric species not only reunites diverged genomes within the same nucleus but also confers aggressive traits that allow the alien species to adapt to local environments (Strong and Ayres, 2013). A similar model of hybridization with adaptation is also widely observed in the other invasive plants [reviewed in Schierenbeck and Ellstrand (2009); Rius and Darling (2014)]. On the other hand, multiple introductions or single origin from various sources offer another strategy to increase the adaptability of IAS (Genton et al., 2005; Kelager et al., 2013). For example, high allelic diversity detected in the introduced populations of garlic mustard (*Alliaria periolata*) is thought to result from multiple introductions (Durka et al., 2005). However, our population genetic and demographic history analyses revealed very recent single origin followed by rapid population expansion of these Chinese populations, indicating that high adaptability of the Chinese populations cannot be explained solely by the above evolutionary mechanisms. Given that all plants collected from the three source US populations were mixed and grown within the same marsh area, we propose that intraspecific genetic admixture might be one of the potential factors acting as a stimulus in the invasion process. In fact, evidence of inter-population mixing has been reported in the other invasive species (Rosenthal et al., 2008; Culley and Hardiman, 2009). In our case, genetic evidence that supports this hypothesis is the observed recombination of private microsatellite alleles in the Chinese population. In addition, similar evidence is further observed in the chloroplast data set that haplotypes of the three source US populations co-occur in the same population. While this kind of genetic evidence is not widely present in all the Chinese populations, it at least suggests the possibility of intraspecific genetic admixture of the three source US populations in the process of invasion. Correlations between the interspecific/intraspecific hybridization and successful invasion have also been reported in other recent studies (Fitzpatrick and Shaffer, 2007; Strong and Ayres, 2009; Keller and Taylor, 2010; Rius and Darling, 2014).

While above analyses demonstrate the importance of intraspecific genetic admixture, the contribution of high genetic diversity harbored in the source US populations cannot be ignored. It has been well documented that high level of genetic diversity shows positive effects on the adaptability of species in responses to varying environments and colonization (Forsman, 2014). As demonstrated in previous experimental study (Wang et al., 2012), high genetic diversity of the invasive *S. alterniflora* populations can not only increase the ability to adapt to local environments, but also reduce the species richness of native species such as *Scirpus mariqueter* (Cyperaceae). In this study, our results illustrate that all the Chinese populations harbor high genetic diversity and show no significant genetic differentiation from the source US populations. In particular, mathematical simulation indicates that sampling size of the three source US populations is enough to represent the genetic constitution of the *S. alterniflora* in China. In addition, an opposite example is the congeneric invasive species *S. anglica* in which low genetic diversity is thought to be a potential factor that has resulted in its rapid decrease in its range in China (An et al., 2004). Taken together, our findings based on multiple microsatellite and chloroplast markers suggest that high genetic diversity of the source US populations followed by genetic admixture of the three independent genetic stocks contributed to the successful establishment of *S. alterniflora* in China. Further investigations by using genome-wide scanning might be able to provide more comprehensive evidence to the successful establishment of *S*. *alterniflora* in China. Specifically, four *Spartina* species (*S. alterniflora*, *S. anglica*, *S. patens*, and *S. cynosuroides*) have been introduced in China and co-occur in natural habitats. It remains a worthwhile issue to examine whether interspecific hybridizations have conferred adaptability to the *S*. *alterniflora*.

## Data Availability Statement

All data sets generated in this study are included in the article/**Supplementary Material**. All DNA sequences can be accessed in GenBank with the accession numbers MK675746–MK675756.

## Author Contributions

LS, L-FL, and BL designed the study. LS, S-YQ, and J-XH performed the experiment. Genetic data analysis was done by LS, with the help of Z-PS and C-CW, and the mathematical simulation was done by YW. The paper was written by LS and L-FL, and revised by Z-PS, LW, JY, C-CW, MN, and BL.

## Funding

This project was financially supported by the National Key Research and Development Program of China (grant no. 2017YFC1200100), National Science Foundation of China (grant no. 41630528 and 31670491), and Shanghai Pujiang Scholar Program (grant no. 19PJ1401500 and 16PJ1400900).

## Conflict of Interest

The authors declare that the research was conducted in the absence of any commercial or financial relationships that could be construed as a potential conflict of interest.
