# Supplementary material for: High Genetic Diversity With Weak Phylogeographic Structure of the Invasive Spartina alterniflora (Poaceae) in China
Source: Front Plant Sci. 2019 Nov 20;10:1467. doi: 10.3389/fpls.2019.01467 (PMC6896949; doi:10.3389/fpls.2019.01467)
Supplement: Supplementary file 1 [file DataSheet_1.pdf]

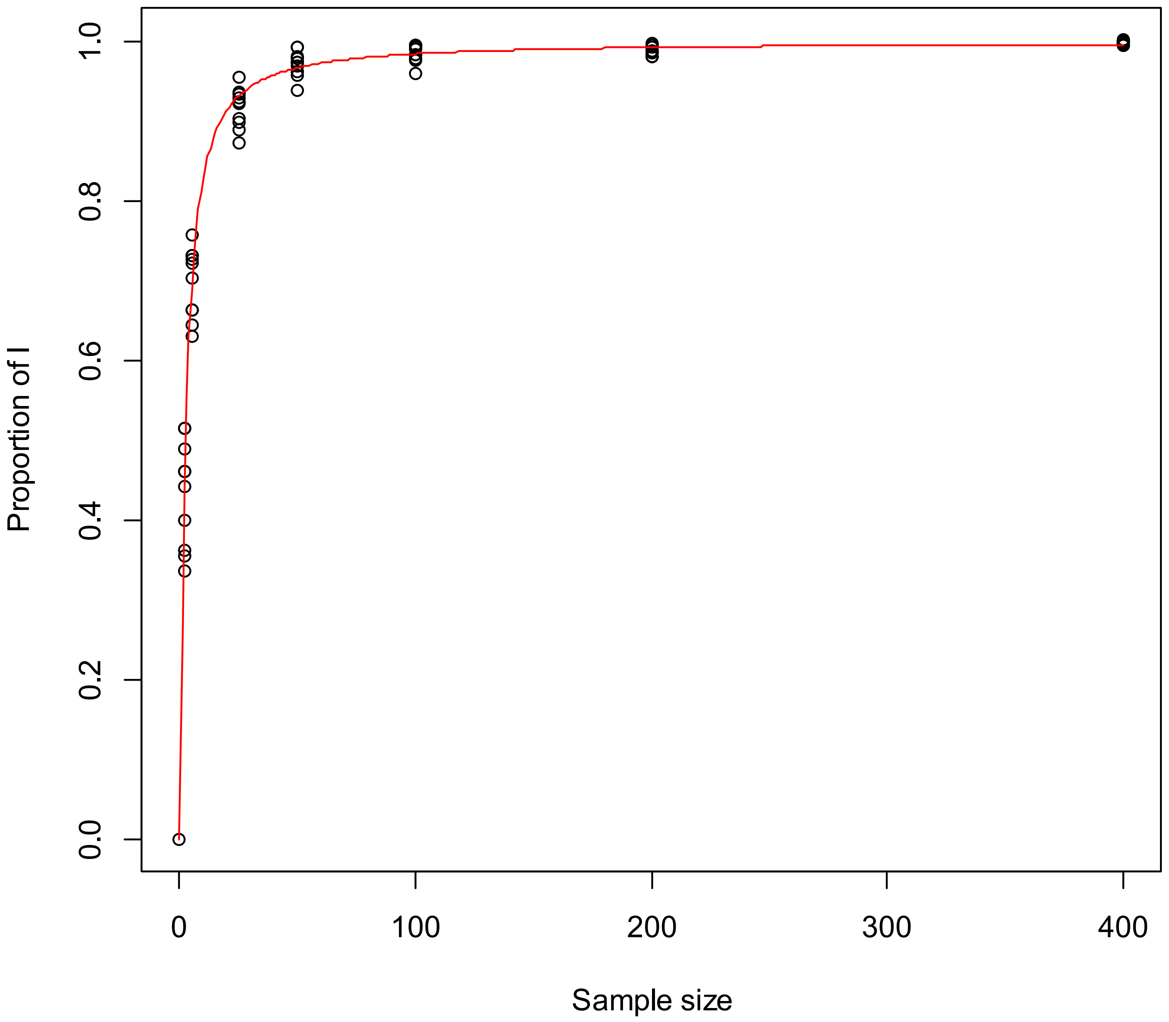

Supplementary Figure 1. The proportion of Shannon's information index (*I*) calculated for different numbers of individuals from US populations. The red curve represents the simulated variation tendency for the proportion of *I* as sample size changes.
