# Supplementary material for: High Genetic Diversity With Weak Phylogeographic Structure of the Invasive Spartina alterniflora (Poaceae) in China
Source: Front Plant Sci. 2019 Nov 20;10:1467. doi: 10.3389/fpls.2019.01467 (PMC6896949; doi:10.3389/fpls.2019.01467)
Supplement: Supplementary file 3 [file DataSheet_3.pdf]

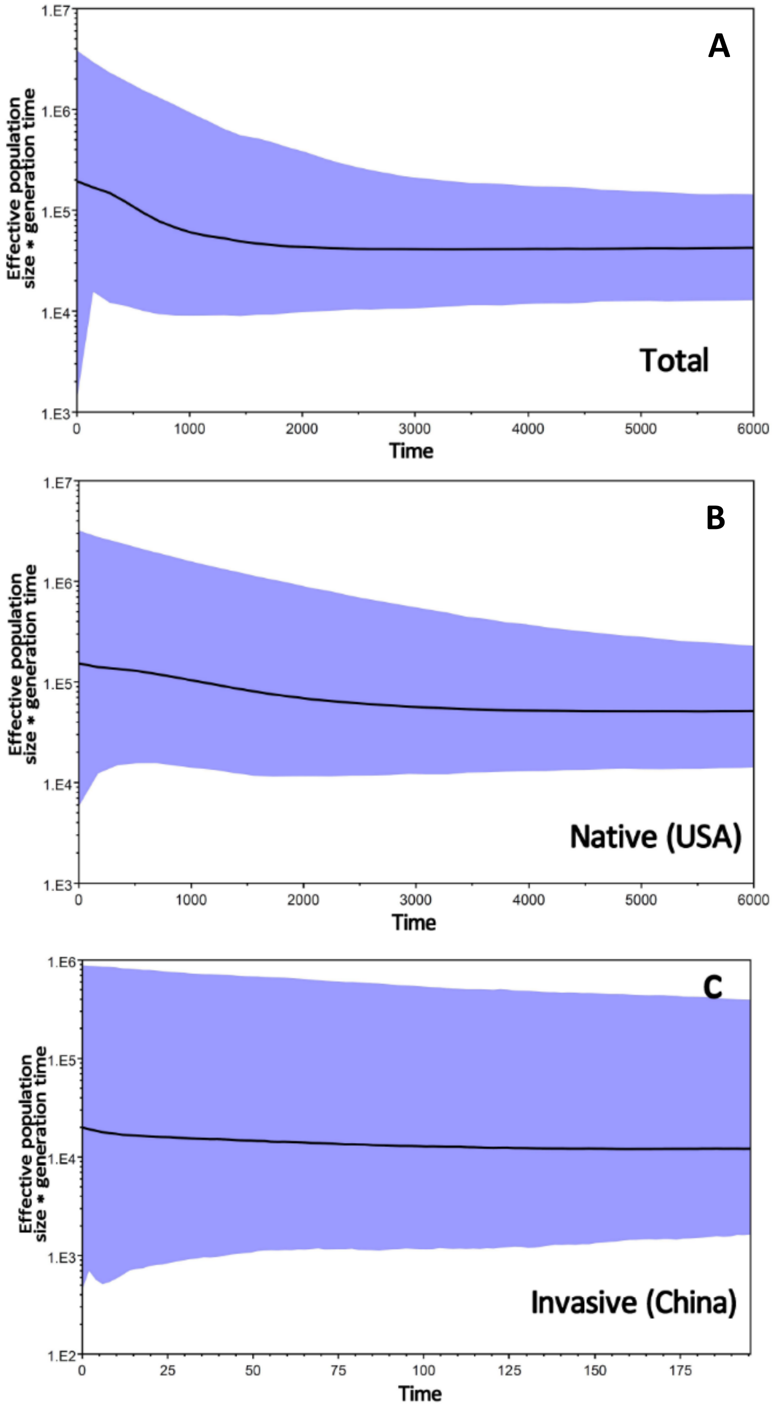

Supplementary Figure 3. Changes of effective population size through time based on Bayesian skyline plot. (A) total populations, (B) native US populations and (C) invasive Chinese populations. Blue areas indicate the 95% confidence intervals.
