# Supplementary material for: High Genetic Diversity With Weak Phylogeographic Structure of the Invasive Spartina alterniflora (Poaceae) in China
Source: Front Plant Sci. 2019 Nov 20;10:1467. doi: 10.3389/fpls.2019.01467 (PMC6896949; doi:10.3389/fpls.2019.01467)
Supplement: Supplementary file 4 [file DataSheet_4.pdf]

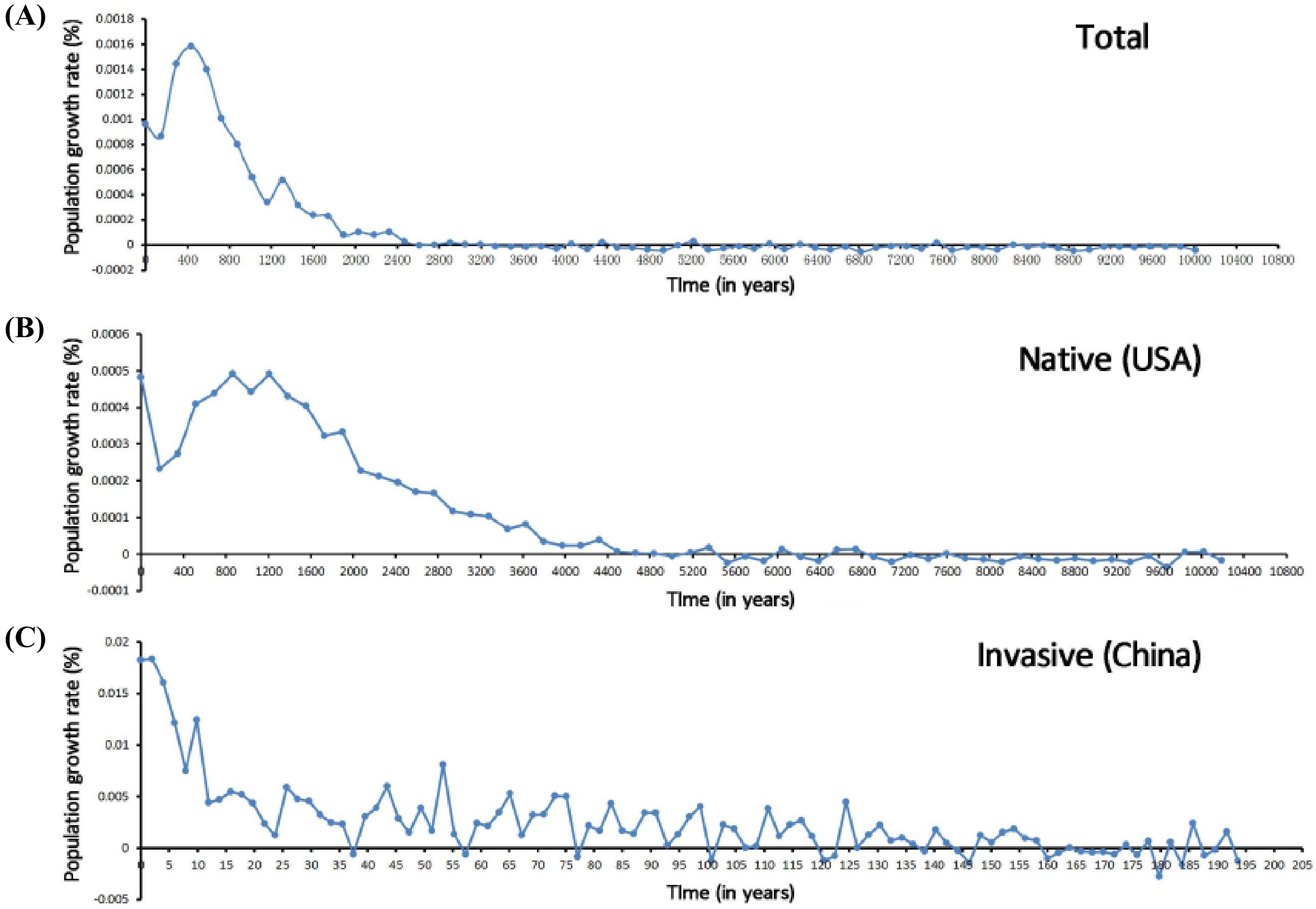

Supplementary Figure 4. Changes of population growth rate through time. (A) total populations, (B) native US populations and (C) invasive Chinese populations.
