# Supplementary material for: High Genetic Diversity With Weak Phylogeographic Structure of the Invasive Spartina alterniflora (Poaceae) in China
Source: Front Plant Sci. 2019 Nov 20;10:1467. doi: 10.3389/fpls.2019.01467 (PMC6896949; doi:10.3389/fpls.2019.01467)
Supplement: Supplementary file 5 [file DataSheet_5.docx]

**Text S1** R codes for simulation of sampling effect for native populations

# Sampling. Take the sample size 100 as an example #

# Data input: genotype data #

a<-read.csv("Gendata.csv")

# Output files definition #

out.name<-paste("out",1:10,".csv",seq="")

# Random sampling for a certain sample size with ten replicates #

for(i in 1:10) {

b<-sample(a$Code,size=100,replace=FALSE,prob=NULL)

c<-a[b,]

write.csv(c,file=out.name[i])

}

# Generation of the fitted curve #

# Data input:the proportion of Shannon's information index (I) for each sample size #

dat<-read.csv("PropofI.csv")

plot(dat,xlab="Sample size", ylab="Proportion of I")

# Nonlinear model #

model<-nls(dat$I~1/(1+dat$N^b),start=c(b=1),data=dat)

# Fitted curve ploting #

x<-0:400

y<-1/(1+x^b)

lines(x,y,col="red")
