# Supplementary material for: High Genetic Diversity With Weak Phylogeographic Structure of the Invasive Spartina alterniflora (Poaceae) in China
Source: Front Plant Sci. 2019 Nov 20;10:1467. doi: 10.3389/fpls.2019.01467 (PMC6896949; doi:10.3389/fpls.2019.01467)
Supplement: Supplementary file 6 [file DataSheet_6.docx]

**Text S2** Computer source codes on LINUX for simulation of population genetic mixing

#include <gsl/gsl_rng.h>

#include <iostream>

#include <fstream>

#include <sstream>

#include <cfloat>

#include <string>

#include <vector>

#include <cmath>

#include <ctime>

#include <omp.h>

using namespace std;

const size_t ploid=2;

const size_t nsim=1000000000ULL;

const size_t startn=20;

const size_t endn=25;

const size_t max_diff=2;

#Variables definition#

class shanglei{

private:

double ba, bb, bc;

inline size_t simulate(size_t N, gsl_rng *R, vector<unsigned> &C);

public:

vector<vector<unsigned> > ca, cb, cc, cd;

vector<vector<double> > fa, fb, fc, fd, fm;

void load_data(const char *N, vector<vector<unsigned> > &C, vector<vector<double> > &F);

void proportion(void);

void estimateN(void);

};

#Data loading: allele frequency #

void shanglei::load_data(const char *N, vector<vector<unsigned> > &C, vector<vector<double> > &F){

ifstream fi(N);

string buf;

while(!fi.eof()){

getline(fi, buf); if(!buf.size()) continue;

vector<unsigned> c; unsigned x, sum=0;

istringstream si(buf);

while(!si.eof()){ x=0xffffffff; si>>x; if(x!=0xffffffff){ c.push_back(x); sum+=x; } }

vector<double> f(c.size());

for(size_t i=0; i<c.size(); i++) f[i]=(double)c[i]/sum;

C.push_back(c); F.push_back(f);

}

fi.close();

}

# Calculation of genetic contribution rates for native source populations #

void shanglei::proportion(void){

double best=-DBL_MAX;

ba=bb=bc=0;

for(double a=0; a<1.0001; a+=0.001) for(double b=0; b<1.0001; b+=0.001){

double c=1-a-b; double chi2=0; if(c<0) continue;

for(size_t i=0; i<fa.size(); i++) for(size_t j=0; j<fa[i].size(); j++){

double f=a*fa[i][j]+b*fb[i][j]+c*fc[i][j], x=fa[i][j]+fb[i][j]+fc[i][j];

if(cd[i][j]&&x){

if(f>0) chi2+=cd[i][j]*log(f);

else chi2=-FLT_MAX;

}

}

if(chi2>best){ best=chi2; ba=a; bb=b; bc=c; }

}

fm.resize(fa.size());

for(size_t i=0; i<fa.size(); i++){

fm[i].resize(fa[i].size());

for(size_t j=0; j<fa[i].size(); j++) fm[i][j]=ba*fa[i][j]+bb*fb[i][j]+bc*fc[i][j];

}

cout<<"proportion:\n";

cout<<"U-MC\t"<<ba<<'\n';

cout<<"U-SI\t"<<bb<<'\n';

cout<<"U-TB\t"<<bc<<'\n';

}

# Simulation of genetic mixing among native source populations #

size_t shanglei::simulate(size_t N, gsl_rng *R, vector<unsigned> &C){

size_t diff=0;

for(size_t i=0; i<cd.size(); i++){

C.assign(cd[i].size(), 0);

vector<double> &a=fa[i], &b=fb[i], &c=fc[i];

size_t na=ba*N+0.5, nb=bb*N+0.5, nc=bc*N+0.5;

for(size_t j=0; j<na; j++){

double ran=gsl_rng_uniform(R), sum=0;

for(size_t k=0; k<a.size(); k++){ sum+=a[k]; if(sum>=ran){ C[k]++; break; } }

}

for(size_t j=0; j<nb; j++){

double ran=gsl_rng_uniform(R), sum=0;

for(size_t k=0; k<b.size(); k++){ sum+=b[k]; if(sum>=ran){ C[k]++; break; } }

}

for(size_t j=0; j<nc; j++){

double ran=gsl_rng_uniform(R), sum=0;

for(size_t k=0; k<c.size(); k++){ sum+=c[k]; if(sum>=ran){ C[k]++; break; } }

}

for(size_t k=0; k<cd[i].size(); k++) if(fm[i][k]){

if((C[k]&&!cd[i][k])||(!C[k]&&cd[i][k])) diff++;

}

if(diff>max_diff) return max_diff+1;

}

return diff;

}

# Estimate of the original sample size (N) for each native source population on the assumption that three native source populations were mixed equally #

void shanglei::estimateN(void){

size_t npro=omp_get_num_procs();

vector<gsl_rng*> rng(npro);

size_t t0=time(NULL);

for(size_t i=0; i<npro; i++){ rng[i]=gsl_rng_alloc(gsl_rng_default); gsl_rng_set(rng[i], t0+i); }

vector<vector<unsigned> > temp(npro);

for(size_t n=startn; n<=endn; n++){

size_t success=0;

#pragma omp parallel for

for(size_t i=0; i<nsim; i++){

size_t k=omp_get_thread_num();

if(simulate(ploid*n, rng[k], temp[k])<=max_diff){

#pragma omp atomic

success++;

}

}

cout<<n<<'\t'<<success<<'\n';

cerr<<n<<'\t'<<success<<'\n';

}

for(size_t i=0; i<npro; i++) gsl_rng_free(rng[i]);

}

# Data output #

int main(int ac, char **av){

if(ac!=5){ cerr<<"shanglei a b c d\n"; return 0; }

shanglei sl;

sl.load_data(av[1], sl.ca, sl.fa);

sl.load_data(av[2], sl.cb, sl.fb);

sl.load_data(av[3], sl.cc, sl.fc);

sl.load_data(av[4], sl.cd, sl.fd);

sl.proportion();

sl.estimateN();

return 0;

}
