# Supplementary material for: High Genetic Diversity With Weak Phylogeographic Structure of the Invasive Spartina alterniflora (Poaceae) in China
Source: Front Plant Sci. 2019 Nov 20;10:1467. doi: 10.3389/fpls.2019.01467 (PMC6896949; doi:10.3389/fpls.2019.01467)
Supplement: Supplementary file 7 [file Table_1.docx]

**Table S1** Geographic locations of each population and the sampling size for the microsatellite and chloroplast genes.

| **Region** | **Site**  **code** | **Sampling site** | **Longitude**  **(°E)** | **Latitude**  **(°N)** | **SSR sample**  **size** | **cpDNA sample**  **size** |
| --- | --- | --- | --- | --- | --- | --- |
| **United** | U-CC(U-NE) | Cape cod, Massachusettes (New England) | -70.2833 | 41.9000 | 38 | 12 |
| **States** | U-RI (U-NE) | Rhode island (New England) | -71.2870 | 41.4881 | 28 |  |
|  | U-LS(U-NE) | Long island sound, New york (New England) | -73.0161 | 40.9507 | 43 |  |
|  | U-MC | Morehead city, North Carolina | -76.8328 | 34.7274 | 48 | 12 |
|  | U-SV | Savannah, Georgia | -81.0911 | 32.0811 | 0 | 2 |
|  | U-SI | Sapelo island, Georgia | -81.2799 | 31.3890 | 90 | 10 |
|  | U-DJ | Dock Junction, Georgia | -81.5069 | 31.2011 | 42 | 8 |
|  | U-TP | Texas point, Texas | -93.8520 | 29.6957 | 83 | 10 |
|  | U-GV | Galveston,Texas | -94.8258 | 29.2811 | 23 | 6 |
|  | U-BR | Brazoria,Texas | -95.5675 | 29.0472 | 24 | 4 |
|  | U-ML | Mosquito lagoon, Florida | -80.8382 | 28.9465 | 0 | 6 |
|  | U-TB | Tampa Bay, Florida | -82.6400 | 27.7731 | 48 | 12 |
|  | U-FP | Fort pierce,Florida | -80.3498 | 27.5362 | 22 | 10 |
| **China** | C-TH | Tanghai, Hebei | 118.1646 | 39.0271 | 97 | 12 |
|  | C-TJ | Tianjin | 117.7544 | 39.0491 | 90 | 12 |
|  | C-DY | Dongying, Shandong | 118.8857 | 38.0194 | 70 | 10 |
|  | C-LY | Lianyungang, Jiangsu | 119.2634 | 34.7700 | 122 | 11 |
|  | C-YC | Yancheng, Jiangsu | 120.6420 | 33.5397 | 67 | 10 |
|  | C-CM | Chongming, Shanghai | 121.9703 | 31.5172 | 39 | 10 |
|  | C-WL | Wenling, Zhejiang | 121.6200 | 28.3501 | 67 | 10 |
|  | C-NH | Hengyu, Fujian | 119.5994 | 26.7032 | 19 | 10 |
|  | C-NF | Feiluan, Fujian | 119.5985 | 26.5869 | 34 | 10 |
|  | C-ZH | Zhuhai, Guangdong | 113.6109 | 22.4143 | 45 | 10 |
|  | C-ZJ | Zhanjiang, Guangdong | 110.1783 | 20.8842 | 76 | 10 |
