# Supplementary material for: High Genetic Diversity With Weak Phylogeographic Structure of the Invasive Spartina alterniflora (Poaceae) in China
Source: Front Plant Sci. 2019 Nov 20;10:1467. doi: 10.3389/fpls.2019.01467 (PMC6896949; doi:10.3389/fpls.2019.01467)
Supplement: Supplementary file 8 [file Table_2.docx]

**Table S2** Microsatellite markers we used in this study

| New code | Marker | Primer sequence (5'-3') | Fluorescence label | Core repeat | Tm (ºC) | Allele size range (bp) | No. of alleles detected |
| --- | --- | --- | --- | --- | --- | --- | --- |
| SP02 | SPAR.02 | F:GAAGGACGAGTCTCATTTGG | 5'FAM | (CT)14 | 60 | 189-203 | 8 |
|  |  | R:GGCTGCCCCTGTTTCACG |  |  |  |  |  |
| SP05 | SPAR.05 | F:AGGTAACACCGAACGAGTC | 5'HEX | (AG)18 | 60 | 190-220 | 16 |
|  |  | R:CCTACGACATCACCGATA | |  |  |  |  |
| SP06 | SPAR.06 | F:CGGTTGTTTTTGATTGTC | 5'HEX | (CT)18 | 55 | 235-259 | 12 |
|  |  | R:GGTTCTTGGGGAGTTGATTTC | |  |  |  |  |
| SP07 | SPAR.07 | F:TTTCATTTCTGCCGCTTTTAC | 5'ROX | (AG)11 | 54 | 275-307 | 17 |
|  |  | R:GTCGCCCCCTAATCTTTCTC | |  |  |  |  |
| SP09 | SPAR.09 | F:GTGGCCTAGCCTATCGACCT | 5'ROX | (CT)12 | 58 | 268-302 | 17 |
|  |  | R:TGAATGGAAAGGGGAAATGA | |  |  |  |  |
| SP10 | SPAR.10 | F:CGCAAAACGAAACCTTGTTC | 5'FAM | (CT)12 | 55 | 334-352 | 10 |
|  |  | R:AGGCTGCTGGACTGACATCT | |  |  |  |  |
| SP11 | SPAR.11 | F:ATTGTCTCCCTCCCTCTTCC | 5'HEX | (CT)12 | 54 | 230-248 | 10 |
|  |  | R:TCATTTCATCGCACTCACG | |  |  |  |  |
| SP20 | SPAR.20 | F:ACCGTGCCTCAGCTACTG | 5'FAM | (GA)10 | 54 | 167-181 | 8 |
|  |  | R:GGTGTTTCCTCGCATAGATC | |  |  |  |  |
| SP27 | SPAR.27 | F:CATCAAAAGCAAGAGGA | 5'ROX | (GA)23 | 54 | 301-345 | 22 |
|  |  | R:GACACCAACGGAACTG |  |  |  |  |  |
