# Supplementary material for: High Genetic Diversity With Weak Phylogeographic Structure of the Invasive Spartina alterniflora (Poaceae) in China
Source: Front Plant Sci. 2019 Nov 20;10:1467. doi: 10.3389/fpls.2019.01467 (PMC6896949; doi:10.3389/fpls.2019.01467)
Supplement: Supplementary file 9 [file Table_3.docx]

**Table S3** Null allele frequency for each population at each locus

| Population | Locus | | | | | | | | | |
| --- | --- | --- | --- | --- | --- | --- | --- | --- | --- | --- |
|  | SP02 | SP05 | SP06 | SP07 | SP09 | SP10 | SP11 | SP20 | SP27 |  |
| U-CC |  |  |  |  | 0.20 |  |  |  | 0.26 |  |
| U-LS |  | 0.11 |  | 0.16 | 0.15 |  |  |  | 0.25 |  |
| U-RI |  | 0.17 | 0.24 | 0.14 | 0.17 | 0.12 |  |  | 0.29 |  |
| U-MC | 0.28 | 0.21 |  | 0.12 |  |  |  |  | 0.23 |  |
| U-SI | 0.20 | 0.25 | 0.13 | 0.13 | 0.16 | 0.05 | 0.07 | 0.11 | 0.23 |  |
| U-DJ | 0.19 | 0.27 |  | 0.11 | 0.27 | 0.13 |  | 0.20 | 0.42 |  |
| U-TP | 0.11 | 0.11 | 0.13 |  |  | 0.06 |  |  | 0.16 |  |
| U-GV |  | 0.15 |  |  | 0.20 | 0.21 |  |  | 0.35 |  |
| U-BR | 0.23 | 0.21 |  |  | 0.08 | 0.15 | 0.20 |  | 0.31 |  |
| U-TB | 0.26 |  | 0.26 |  |  |  | 0.15 |  |  |  |
| U-FP | 0.18 |  |  |  |  |  | 0.23 |  | 0.36 |  |
| C-TH | 0.18 |  |  | 0.13 |  |  | 0.08 |  | 0.28 |  |
| C-TJ | 0.14 | 0.11 | 0.06 | 0.12 |  |  |  | 0.07 | 0.28 |  |
| C-DY | 0.28 | 0.10 |  |  | 0.12 |  | 0.08 | 0.14 | 0.40 |  |
| C-LY | 0.30 | 0.17 | 0.06 | 0.06 |  |  | 0.12 |  | 0.25 |  |
| C-YC | 0.17 | 0.22 |  | 0.09 |  |  |  |  | 0.25 |  |
| C-CM | 0.23 |  |  |  | 0.16 | 0.17 |  | 0.10 | 0.40 |  |
| C-WL |  | 0.10 |  |  |  |  |  |  | 0.32 |  |
| C-NH |  |  | 0.14 |  |  | 0.09 |  |  | 0.36 |  |
| C-NF |  |  |  |  |  |  |  |  | 0.36 |  |
| C-ZH | 0.19 | 0.26 |  |  |  |  |  |  |  |  |
| C-ZJ |  |  |  | 0.13 |  |  |  |  | 0.34 |  |
