# Supplementary material for: High Genetic Diversity With Weak Phylogeographic Structure of the Invasive Spartina alterniflora (Poaceae) in China
Source: Front Plant Sci. 2019 Nov 20;10:1467. doi: 10.3389/fpls.2019.01467 (PMC6896949; doi:10.3389/fpls.2019.01467)
Supplement: Supplementary file 10 [file Table_4.docx]

**Table S4** Variable sites detected in the *trnT2*-*rps4*, *trnT*-*trnL* and *rbcL*-*psaI* intergenic spacer of *S. alterniflora*, identifying 10 haplotypes.

| **Haplotype** | **trnT2-rps4 spacer 545bp** | | |  | **trnT-trnL spacer 502bp** | | |  |  | **rbcL-psaI spacer 748bp** | | |
| --- | --- | --- | --- | --- | --- | --- | --- | --- | --- | --- | --- | --- |
|  | **55** | **70** | **535** |  | **90** | **117** | **474** |  |  | **84** | **490** | **569** |
| H1 | C | C | C |  | T | A | T |  |  | T | C | A |
| H2 | C | C | C |  | T | A | T |  |  | G | T | T |
| H3 | C | N | C |  | T | A | T |  |  | G | C | T |
| H4 | C | C | C |  | T | A | T |  |  | G | C | T |
| H5 | C | N | C |  | G | A | G |  |  | G | T | T |
| H6 | C | N | C |  | T | A | G |  |  | G | T | T |
| H7 | T | N | C |  | T | A | G |  |  | G | C | T |
| H8 | C | N | C |  | T | A | G |  |  | G | C | T |
| H9 | C | N | N |  | T | A | G |  |  | G | C | T |
| H10 | C | C | C |  | T | T | T |  |  | T | C | A |
