# Supplementary figures and images for: High Genetic Diversity With Weak Phylogeographic Structure of the Invasive Spartina alterniflora (Poaceae) in China
Source: Front Plant Sci. 2019 Nov 20;10:1467. doi: 10.3389/fpls.2019.01467 (PMC6896949; doi:10.3389/fpls.2019.01467)

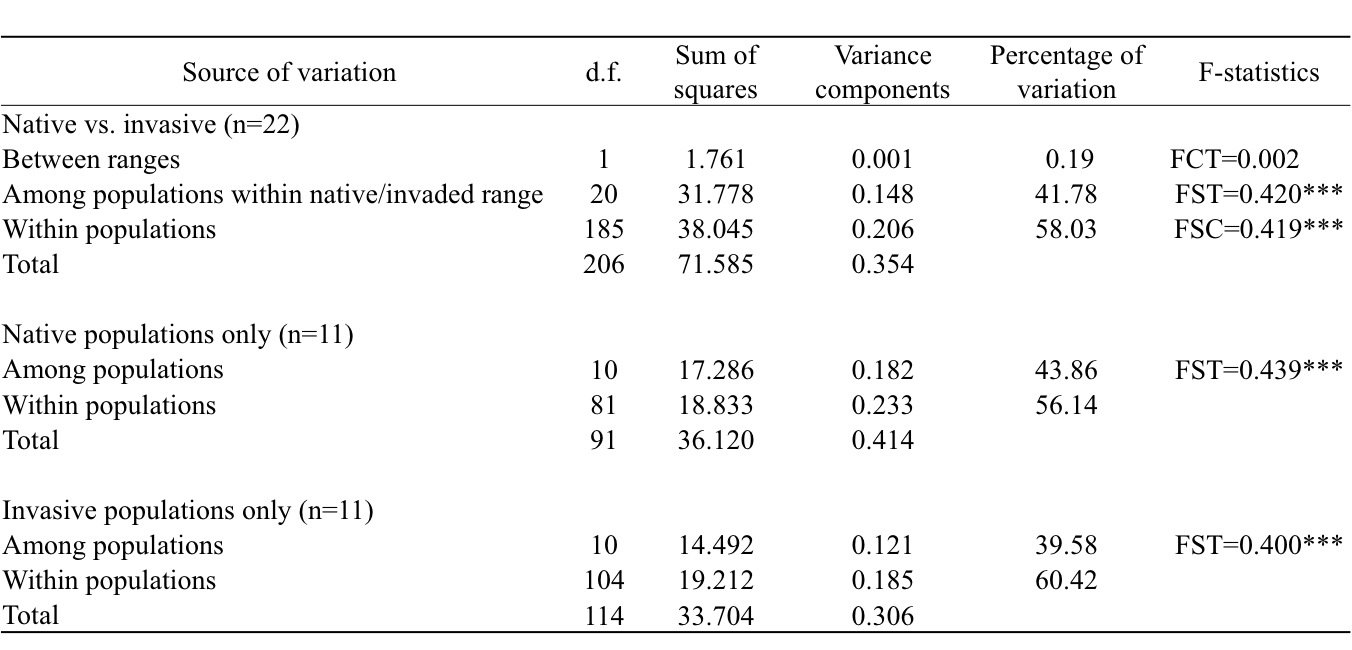
**Table S5** Analysis of molecular variance for populations of *S. alterniflora*

Supplement: Supplementary file 11 [file Table_5.docx]
