# Supplementary material for: High Genetic Diversity With Weak Phylogeographic Structure of the Invasive Spartina alterniflora (Poaceae) in China
Source: Front Plant Sci. 2019 Nov 20;10:1467. doi: 10.3389/fpls.2019.01467 (PMC6896949; doi:10.3389/fpls.2019.01467)
Supplement: Supplementary file 12 [file Table_6.docx]

| **Table S6. Detail of simulation results based on Approximate Bayesian Computation (ABC).** | | | | | | | | | | |
| --- | --- | --- | --- | --- | --- | --- | --- | --- | --- | --- |
| Sample size | Match frequency | Sample size | Match frequency | Sample size | Match frequency | Sample size | Match frequency | Sample size | Match frequency |  |
|  | (per billion) |  | (per billion) |  | (per billion) |  | (per billion) |  | (per billion) |  |
| 1 | 0 | 27 | 0 | 53 | 127 | 79 | 207 | 105 | 50 |  |
| 2 | 0 | 28 | 0 | 54 | 135 | 80 | 223 | 106 | 39 |  |
| 3 | 0 | 29 | 0 | 55 | 134 | 81 | 205 | 107 | 46 |  |
| 4 | 0 | 30 | 0 | 56 | 145 | 82 | 190 | 108 | 44 |  |
| 5 | 0 | 31 | 1 | 57 | 195 | 83 | 212 | 109 | 39 |  |
| 6 | 0 | 32 | 2 | 58 | 222 | 84 | 183 | 110 | 37 |  |
| 7 | 0 | 33 | 2 | 59 | 228 | 85 | 203 | 111 | 34 |  |
| 8 | 0 | 34 | 1 | 60 | 239 | 86 | 169 | 112 | 32 |  |
| 9 | 0 | 35 | 5 | 61 | 276 | 87 | 196 | 113 | 23 |  |
| 10 | 0 | 36 | 4 | 62 | 242 | 88 | 126 | 114 | 18 |  |
| 11 | 0 | 37 | 10 | 63 | 170 | 89 | 107 | 115 | 16 |  |
| 12 | 0 | 38 | 8 | 64 | 187 | 90 | 114 | 116 | 9 |  |
| 13 | 0 | 39 | 4 | 65 | 197 | 91 | 80 | 117 | 17 |  |
| 14 | 0 | 40 | 11 | 66 | 178 | 92 | 81 | 118 | 11 |  |
| 15 | 0 | 41 | 14 | 67 | 179 | 93 | 87 | 119 | 8 |  |
| 16 | 0 | 42 | 15 | 68 | 203 | 94 | 68 | 120 | 7 |  |
| 17 | 0 | 43 | 18 | 69 | 196 | 95 | 71 | 121 | 9 |  |
| 18 | 0 | 44 | 24 | 70 | 213 | 96 | 66 | 122 | 7 |  |
| 19 | 0 | 45 | 40 | 71 | 221 | 97 | 70 | 123 | 4 |  |
| 20 | 0 | 46 | 44 | 72 | 184 | 98 | 67 | 124 | 7 |  |
| 21 | 0 | 47 | 52 | 73 | 213 | 99 | 51 | 125 | 7 |  |
| 22 | 0 | 48 | 60 | 74 | 211 | 100 | 53 | 126 | 4 |  |
| 23 | 0 | 49 | 77 | 75 | 214 | 101 | 60 | 127 | 9 |  |
| 24 | 0 | 50 | 71 | 76 | 226 | 102 | 58 | 128 | 8 |  |
| 25 | 0 | 51 | 88 | 77 | 200 | 103 | 56 | 129 | 2 |  |
| 26 | 0 | 52 | 111 | 78 | 202 | 104 | 53 | 130 | 9 |  |
